# Deep Reads: How I learnt to love population genetics

**DOI:** 10.1371/journal.pgen.1007140

**Published:** 2017-12-21

**Authors:** Jonathan Flint

**Affiliations:** Center for Neurobehavioral Genetics, University of California Los Angeles, Los Angeles, California, United States of America

**Fig 1:**
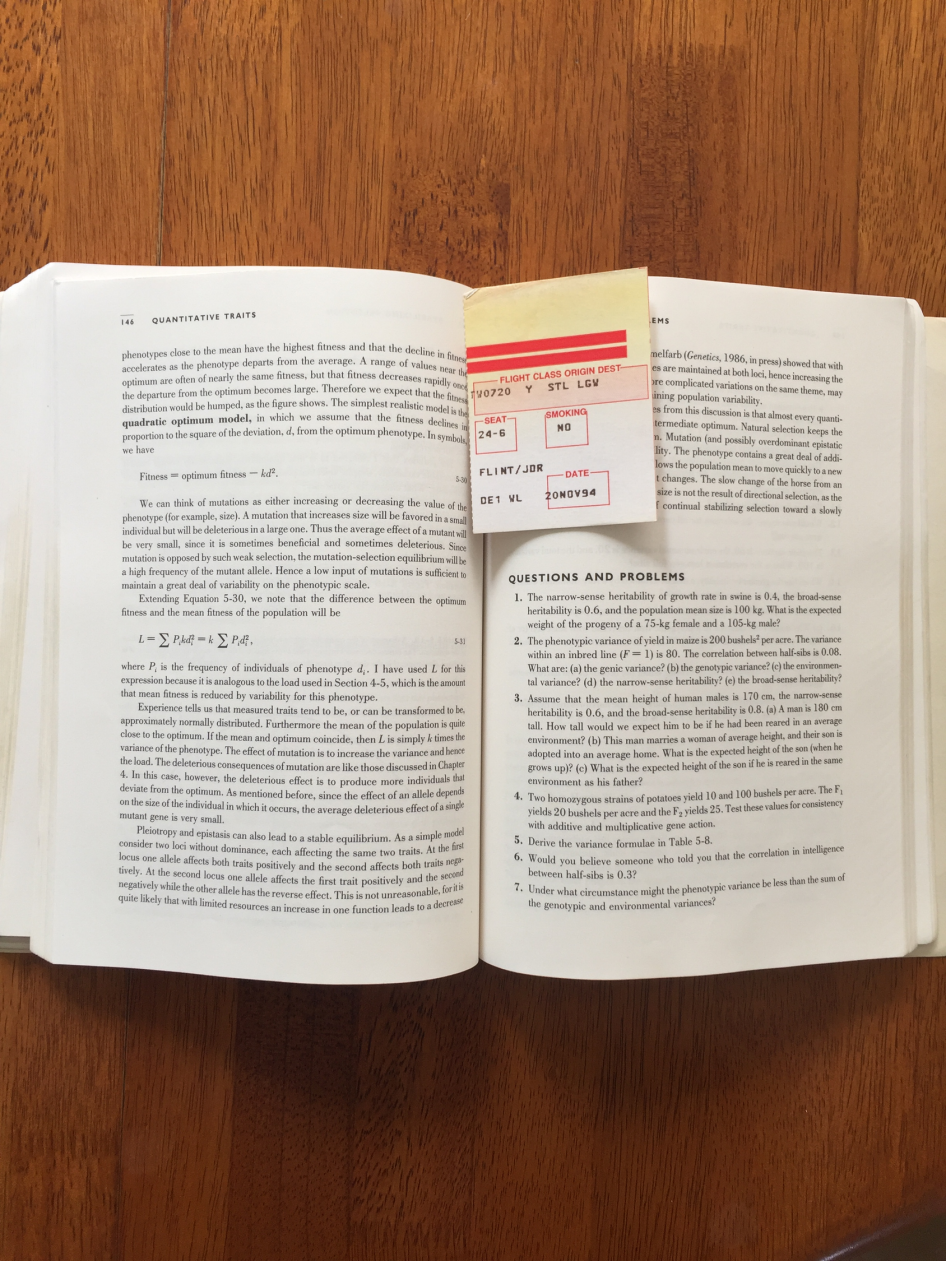
My copy of Crow's book (Chapter 5). Image courtesy of Jonathan Flint.

I’m not sure when I bought my copy of James F. Crow’s “Basic Concepts in Population, Quantitative and Evolutionary Genetics” [1] but it could not have been too long after the first edition came out in 1986 (there is a stub of a plane ticket in my copy dated 1994, so I must have bought it before then; for a good many years around that time the book was a comfort item for me; Fig 1). It certainly wasn’t the sort of book I would have bought by choice, as most of the contents are devoted to a subject I really don't like much (population genetics), but I needed an introduction to quantitative genetics and this was recommended to me by a senior colleague.

In many ways it is an old-fashioned text book; there are none of those boxes, in-depth analyses, short bios of famous scientists, and other features beloved of publishers wishing to make a book more accessible, and hence to increase sales. No, this is a proper textbook. It has tables, and black and white figures, and lots of text. But it’s a short textbook, less than 250 pages long, a great advantage in my view, since I wasn’t looking forward to dealing with the material. I had a background in molecular biology, and the only formal training I had ever had in genetics consisted of some lectures from a medical geneticist, focused primarily on what could be done for families in which a Mendelian disorder was segregating (answer: not much).

I wanted to find out what was known about the genetic basis of quantitative traits, things like height and weight, or, more relevant since I worked in behavior genetics, measures of anxiety in mice. At the time I bought the book, I was about to start an experiment that involved crossing inbred mice whose ancestors had been selected for many generations on a behavioral phenotype, activity in an open-field arena. A series of classic papers in the behavior genetics literature from John DeFries described the selection experiments and discussed their implications for the genetic basis of behavior. DeFries had already used crosses between mice to identify the effect of a single gene on open field activity [2] and I wanted to take those findings into the molecular age, map the genes, and discover the molecular mechanisms. I knew nothing about quantitative genetics, but of course that didn’t stop me thinking I could soon make an important contribution. I decided to educate myself enough to understand what the previous generation of behavior geneticists had accomplished, but I was pretty certain we’d move on quickly to the molecular stuff, you know the sort of thing that the editors of Cell like, so that other concerns (like population genetics) would soon be left far behind.

While Crow’s book did have one chapter (Chapter 5) entitled “Quantitative traits”, I admit I was disappointed when I looked over the contents list. Crow doesn’t devote much space to molecular biology, which didn’t impress me much, since after all it was blindingly obvious (to me, then) that the newly introduced molecular approaches would soon solve all the mysteries of genetics. Did I really need all those chapters on inbreeding, migration, population structure and selection? I rushed straight to Chapter 5. It started off badly. Just a couple of pages in, I read “The long-range aim of such research is to find what each allele is doing and how it interacts with each of the other alleles. Although molecular analysis will eventually clarify many examples, at present it is impractical and for many purposes unimportant”. What? Unimportant? Why had I a bought a book by so ill-informed an author? Worse was to follow. At the top of the next page I read “for natural selection to work, gene action must exhibit a certain predictability”. I had no intention of learning about selection! The book had cost me £15.95 –quite an expense for me at that time. Had I wasted my money?

I went back to the beginning of the book, and found to my surprise, a ringing endorsement of the value of molecular techniques! “The inheritance of most molecular traits… follows simple Mendelian rules, so with increasing molecular study, simple theory becomes more applicable…Population genetics includes a large body of mathematical theory, one of the richest and most successful in biology. The useful application of this theory has been greatly enhanced in recent years by an abundance of new molecular techniques”. This was more like it! I liked “abundance of new molecular techniques”; that was certainly a good thing. But there was that troubling reference to the mathematical theory of population genetics, something I really wasn’t intending to come to grips with.

Population genetics, for those of you unfamiliar with this field, has for its subject matter to decide upon the relative importance of four factors: selection, migration, mutation and something called “genetic drift” (this refers to the random fluctuation of alleles in the absence of the other three factors, a sort of “one minus” factor, as in “minus the effect of selection, migration and mutation”). Presented with a measurement of the frequency of your favourite allele in a population (something that might have taken you years to estimate) a population geneticist explains to you that the reason 45% of the population carry the allele is because of selection, migration, mutation and genetic drift. Great, really helpful. As far as I could tell, population geneticists had no way of ever really deciding which, if any, of these factors was responsible, because no population geneticist ever carried out an experiment. All they did was analyze data collected from natural populations (don't tell anyone, but while I am a little more appreciative of the ingenuity brought to bear on problems in population genetics, I still think those statements about the field are broadly true).

There isn’t really an introduction to Crow’s book. Apart from a short preface, he doesn’t waste any time before getting into the meat of his subject. By page six he’s describing randomly mating populations and the Hardy-Weinberg principle. *That* was something I knew about; departures from Hardy-Weinberg were useful in detecting errors in the way genotypes had been called, and I had used them as part of quality control. Imagine my surprise when I read: “Despite its simplicity, the Hardy-Weinberg principle forms the basis for almost all analysis of natural diploid populations. The single-generation approach to equilibrium and the approximate random mating that characterizes most populations ensure its applicability”. How could that be true, and what on earth did he mean by “single-generation approach to equilibrium”? I stopped and went back to read in more detail. The Hardy-Weinberg principle states that the frequency of genotypes in a population can be predicted from the frequency of alleles as a binomial distribution. That is, if there are two alleles in a population with frequency p and q (where p + q = 1), the homozygotes will have frequencies p^2^ and q^2^ and the heterozygotes 2pq; in other words, the expansion of (p+q)^2^.

Crow claimed that Hardy-Weinberg ratios are attained in a single generation of random mating. He did more than claim. On page 9 he lays out exactly why random choice from a pool of gametes (a big bag of ps and qs) to create genotypes is the same as the production of genotypes from the random mating of a large population of diploid individuals. I hadn’t realized that, but then, as I was beginning to realize, I hadn’t thought much about this subject. I certainly hadn’t thought about it mathematically. Later in the chapter (page 23) was a good example of why I should have paid more attention. By this point Crow (still moving fast) has covered two locus models, and described linkage disequilibrium, another term I thought I was familiar with. He describes allele frequencies at blood group loci obtained from a population in Michigan. Each locus showed Hardy-Weinberg equilibrium, consistent with expectations, but the two locus analyses do not. In fact, some pairs of loci situated on different chromosomes were in disequilibrium! How was that possible?

Random mating drives loci to Hardy-Weinberg ratios in one generation, as I now knew, but that’s not true for gametic equilibrium (where genotypes are made up of alleles at two or more loci). Suppose there are two loci with two alleles (A and a, B and b). That gives a total of ten genotypes and the frequencies of each are the products of the frequencies of each component, so the homozygote AB is P^2^_AB_, and so on (see [1], Table 1–12). But that calculation assumes the frequencies are independent. If the two loci are close to each other on a chromosome, then the genotypes won’t be independent. Migrants from different parts of the world make up a population like that of Michigan, bringing with them alleles at very different frequencies. There hasn’t been enough time for random mating to result in equilibrium. This insight was obtained with mathematics even I could understand. I began to treat the book with more respect.

I also began to notice another characteristic. In a slightly diffident tone, Crow would often introduce the work of other authors to his readers, along the lines of “here’s something you might enjoy”. The suggestion that I might enjoy any work by a mathematician was totally novel, but I was intrigued by Crow’s brief history of the Hardy-Weinberg equilibrium, “The principle is a simple application of the binomial theorem; so almost anyone who considered the problem might have discovered it. Hardy was one of the world’s greatest mathematicians; you might enjoy his charming but idiosyncratic essay “A Mathematician’s Apology” (1940) [3]. “Simple application”? Almost anyone might have discovered it? Well, not me for sure. I found Crow’s recommendation hard to believe, but I did get a copy of Hardy’s book. Astonishingly, Crow was right! It *is* a good read.

I was taking Crow more seriously now, and began to experience a sinking feeling as I confronted the extent of my ignorance. I think either Francis Crick or Sydney Brenner (probably both) counseled against reading, as it inhibits thought. I can see their point. Reading Crow was beginning to terrify me. Taking the book seriously meant not only reading the text but (scary, scary) also answering the questions at the end of each chapter. “In a population there are 10 times as many MN as NN genotypes. What is the frequency of the N allele?” Oh, I know that! Must be an application of the Hardy-Weinberg principle. So 2P_MN_ must be equal to 10P^2^_N_, and P_M_ = 5P_N_, and since P_M_ + P_N_ are always equal to 1, P_N_ must equal 1/6. That wasn’t so bad. This cheered me up. Actually, I could answer most of the questions at the end of the chapter (there are 25 of them and if you get really stuck you can cheat! He gives you the answers at the back of the book).

Back to Chapter 5 and quantitative traits. There was some stuff on selection, including a figure of changes in mouse size (though sadly not mouse open-field activity), and some (not very interesting to me) arguments about multiplicative versus additive action and then, suddenly, at the beginning of section 5–2 this resplendent sentence: “Much of quantitative genetics is concerned with understanding regression and predicting the results of selection when regression occurs”. What on earth did he mean? As far as I knew, regression was some technical thing that statisticians used, what had it to do with quantitative genetics? There is a very fine diagram (Figure 5–5 in my edition) that explains regression to the mean. It means that the children of parents at the extremes of a distribution are more likely to have scores that are closer to the average. I remembered something slightly upsetting. I had recently met Hans Eysenck, then one of the world’s most famous psychologists, at a flat in South London, close to the Institute of Psychiatry where he used to work. Eysenck pulled off the bookshelves a copy of his autobiography. I opened it and idly glanced at the dedication page, which read something like: “To my children. May regression to the mean not affect them too severely”. Oh, now I knew what that meant. I wondered if his children had read it and if they understood too. I wondered, also, could knowledge of regression to the mean be a comfort for all those high achieving parents, whose children don’t get into the best universities?

Chapter 5 introduced me to another of Crow’s teaching techniques. Understanding regression to the mean requires some knowledge of variance and co-variance. In his usual lapidary style Crow gives “the principal statistical measures used in quantitative genetics”–clearly that was something I needed to know. Crow, I realized, was being kind to his readers. He includes the formulae both in an Appendix and in the main text. So far I hadn’t bothered with the appendix. I had a quick look. To my astonishment the appendix is a self-contained description of probability and statistics. Crow takes nothing for granted. He begins with “Definition of Probability”. What could be a better, more reassuring place to begin than that? Yet, in only 30 pages he goes from defining the probability of an event, to functional invariance, a property of maximum likelihood estimates. The introduction to maximum likelihood starts “The logic of this method is simple…” and, yes, there is an equation that demands you know what differential calculus looks like, but even if you don’t, he provides a figure that makes the example clear. The explanatory text is a model of succinct clarity.

I have to admit I have never worked through all the chapters in Crow’s book in detail. It’s worth reading the short preface before you dive in: “Chapters 3 and 6 are the most difficult ones in the text. Chapter 6 demands more facility with algebra and calculus than the rest of the book. You can skip these chapters without loss of continuity if you want a brief introduction to the material usually included in a population and quantitative genetics course”. I took his advice. But one thing that should not be skipped, are the Questions and Problems at the end of each chapter. Yes, I know, it is scary, especially when you think you have understood the concepts and then find you can’t answer a problem, but take it from me, there are some golden moments hidden at the back of each chapter. Crow isn’t just asking you to solve equations and derive formulae (though there is some of that). No, his questions often go further. Let me give just one example. Question 11, chapter 5 (chapter 5 is my favourite chapter and I admit I know it better than the rest of the book): “What is the heritability of sex?” Reading that pulled me up short. How *could* sex be heritable? But then of course it must be heritable, since there is a genetic variant for it. Heritability comes in two forms, broad sense and narrow (or additive) heritability. Additive heritability measures the effects of regression, the joint effect of alleles that contribute to resemblance between siblings (that’s why regression is so central to quantitative genetics). While the broad sense heritability of sex is 100%, the additive contribution must be zero, since knowing the sex of one child is no predictor of a sibling’s sex.

Crow’s book is not the only textbook on quantitative genetics. I might have started with Douglas Falconer’s classic Introduction to Quantitative Genetics (1960) (no mention of population genetics there!) [4]. I came across Falconer’s book (in the excellent edition that has Trudy Mackay as a co-author [5]) later. However, by then I was long married to Crow’s text, and I still won’t consider a divorce. Let me give you a taste of the difference. Here is Falconer, in his best style, introducing quantitative traits: “It will be obvious, to biologist and layman alike, that the sort of variation discussed in the foregoing chapters embraces but a small part of the naturally occurring variation. One has only to consider one’s fellow men and women to realize that they all differ in countless ways, but that these differences are nearly all matters of degree and seldom present clear-cut distinctions attributable to the segregation of single genes. If, for example, we were to classify individuals according to their height, we could not put them into groups labeled “tall” and “short,” because there are all degrees of height, and a division into classes would be purely arbitrary. Variation of this sort without natural discontinuities, is called continuous variation, and characters that exhibit it are called quantitative characters or metric characters, because their study depends on measurement instead of on counting. The genetic principles underlying the inheritance of metric characters are basically those outlined in the previous chapters, but since the segregation of the genes concerned cannot be followed individually, new methods of study have had to be developed and new concepts introduced”.

Crow, with almost the same number of words, does a different job in introducing quantitative variation. “Most of our knowledge about inheritance comes from the study of traits determined by either single allelic differences or a small number of differences. Part of Mendel’s genius was his choice of clear-cut traits; it was his good luck that they turned out monogenic and not closely linked. The success of classical genetics in mutation analysis and chromosome mapping, as well as the spectacular achievements of molecular genetics, bear testimony to the power of genetic methodology using well-chosen marker loci. Quantitative genetics, on the other hand, is concerned with differences of degree rather than kind. Phenotypes are classified not by the presence or absence of certain characteristics, for example, a gel band or a clear phenotype, such as single bristles in Drosophila, but as values measured in centimeters, pounds or bushels. Although the trait sometimes does not lend itself to direct measurements, individuals can be ranked. We can say that Joe is a better geneticist than John, but to say he is twice as good makes no sense”.

Their styles are so different. Falconer’s use of the impersonal pronoun “one”, contrasts with Crow’s direct statements. I love Crow’s last sentence. I hope you can still find a copy and read more stuff like that. But if you can’t find one, then let me make one thing clear. You are not borrowing mine.
